# Advancing semantic segmentation: Enhanced UNet algorithm with attention mechanism and deformable convolution

**DOI:** 10.1371/journal.pone.0305561

**Published:** 2025-01-16

**Authors:** Effat Sahragard, Hassan Farsi, Sajad Mohamadzadeh

**Affiliations:** Department of Electrical and Computer Engineering, University of Birjand, Birjand, Iran; Institut de Robotica i Informatica Industrial, SPAIN

## Abstract

This paper presents a novel method for improving semantic segmentation performance in computer vision tasks. Our approach utilizes an enhanced UNet architecture that leverages an improved ResNet50 backbone. We replace the last layer of ResNet50 with deformable convolution to enhance feature representation. Additionally, we incorporate an attention mechanism, specifically ECA-ASPP (Attention Spatial Pyramid Pooling), in the encoding path of UNet to capture multi-scale contextual information effectively. In the decoding path of UNet, we explore the use of attention mechanisms after concatenating low-level features with high-level features. Specifically, we investigate two types of attention mechanisms: ECA (Efficient Channel Attention) and LKA (Large Kernel Attention). Our experiments demonstrate that incorporating attention after concatenation improves segmentation accuracy. Furthermore, we compare the performance of ECA and LKA modules in the decoder path. The results indicate that the LKA module outperforms the ECA module. This finding highlights the importance of exploring different attention mechanisms and their impact on segmentation performance. To evaluate the effectiveness of the proposed method, we conduct experiments on benchmark datasets, including Stanford and Cityscapes, as well as the newly introduced WildPASS and DensPASS datasets. Based on our experiments, the proposed method achieved state-of-the-art results including mIoU 85.79 and 82.25 for the Stanford dataset, and the Cityscapes dataset, respectively. The results demonstrate that our proposed method performs well on these datasets, achieving state-of-the-art results with high segmentation accuracy.

## 1. Introduction

In image processing, advanced techniques and deep learning methods have shown remarkable improvements in various tasks such as pattern recognition, person re-identification, object detection, image restoration, image classification and image segmentation [1–4]. Image segmentation is a fundamental task in computer vision that involves dividing an image into multiple regions or segments. It plays a crucial role in applications like object recognition, scene understanding, medical imaging, image captioning, and autonomous driving [5–7]. Traditional methods for image segmentation rely on handcrafted features and may not capture the full visual information in complex images. These methods have limitations in terms of their expressive power and handling complex image segmentation tasks [8].

Image segmentation typically involves three steps: pre-processing, feature extraction, and clustering. Pre-processing enhances image quality by removing noise and artifacts, while feature extraction identifies relevant features that distinguish between different regions. Clustering then groups pixels with similar characteristics into segments. Traditional semantic segmentation methods rely on low-level features such as color, shape, and texture, but these approaches are limited in capturing complex visual information [9]. Recent advancements in deep learning, particularly convolutional neural networks (CNNs), have revolutionized semantic segmentation [10]. CNN-based techniques can learn features from raw pixel data and produce pixel-level predictions, capturing intricate pixel-level relationships and leading to more precise and reliable segmentation results [11]. Various strategies, such as image pyramids, encoder-decoder frameworks [12, 13], and context modules [14], have been utilized to improve segmentation performance. Additionally, techniques like spatial pyramid pooling (SPP) [15], deformable convolution [16] and attention mechanisms [17–20] have shown promising results in enhancing segmentation quality. The reported method [21] highlights for the first-time large kernels in image segmentation. However, instead of incorporating extra-large kernels to capture long-range dependencies, the paper employed a refinement module to directly extract features from large kernels. The idea was further expanded by [22]. By re-parameterization, the kernels were enlarged to 31×31. The authors propose RepLKNet, a pure CNN architecture whose kernel size is as large as 31×31, in contrast to commonly utilized 3×3. RepLKNet achieves comparable or superior results on ImageNet and a few typical downstream tasks with lower latency. Yang and colleagues [19] employed large kernels to enhance the effectiveness of spatial pyramid pooling and showcased its benefits in the road extraction task. Additionally, several other studies, such as LKASR [23] for lightweight image super-resolution and LKD-Net for single image dehazing, also incorporated large kernels as attention mechanisms. The UNet architecture, which combines encoders and decoders, has been widely used for semantic segmentation due to its ability to capture both local and global contexts [24, 25]. Atrous Spatial Pyramid Pooling (ASPP) is a module used in semantic segmentation that samples a feature layer at different rates to capture objects and contextual information at multiple scales. Instead of directly resampling features, ASPP utilizes parallel atrous convolutional layers with varying sampling rates to achieve this mapping efficiently [26].

However, the traditional UNet model has limitations in capturing fine details, handling multi-scale data, and modeling long-range interactions. These limitations can affect the accuracy of segmentation results, particularly in complex scenarios. In this paper, we present an advanced method to address the limitations of the traditional UNet model and improve semantic segmentation performance. Our approach integrates deformable convolution, attention ASPP, and attention mechanisms on the decoder path of UNet architecture. Deformable convolution enhances the network’s capability to identify object boundaries and detect complex structures by capturing fine-grained details [16]. The attention ASPP module facilitates robust multi-scale context modeling, improving segmentation accuracy. Additionally, attention mechanisms, specifically the Large Kernel Attention (LKA) [21] module, refine feature representations and focus on relevant regions. This paper contributes in three parts:

1. Integration of Deformable Convolution in the UNet Architecture: We propose the incorporation of deformable convolution in the UNet architecture to capture fine-grained details and enhance the distinction of object boundaries. By leveraging deformable convolutions, our method improves the representation of features and achieves better segmentation accuracy.
2. Introducing Attention ASPP Module for Context Modeling: To enable effective context modeling at multiple scales, we introduce an attention ASPP module. This module incorporates attention mechanisms and considers various scales of contextual information. By integrating attention mechanisms, our approach enhances the precision of segmentation results and captures more semantic details.
3. Utilization of Attention Mechanisms for Feature Refinement: In the decoding path of the UNet, we explore the integration of attention mechanisms, specifically the LKA (Large Kernel Attention) module. By combining low-level and high-level features and applying LKA, we refine feature representations to focus on relevant regions and improve discrimination of object classes.

In the subsequent sections, we review the current literature on semantic segmentation techniques, present our proposed methodology, evaluate its performance on benchmark datasets, and conclude with directions for future research.

## 2. Related work

Over the past decade, deep learning has revolutionized various domains, including machine vision [25], object tracking [26], and segmentation [10]. These techniques enable automatic learning from large datasets and meaningful representation extraction. In the context of semantic segmentation, there have been notable advancements, and this literature review aims to provide a comprehensive understanding of the achievements, limitations, and recent developments in the field. The foundational method, Fully Convolutional Network (FCN) [27], introduced end-to-end pixel-wise segmentation but struggles with capturing fine details and object boundaries in urban images. Several approaches have been proposed to address these limitations, such as replacing the backbone of FCN with ResNet50 [28], which significantly improves segmentation accuracy. UNet [29] and its enhanced version, UNet++ [30], introduced an encoder-decoder structure with skip connections to accommodate scale variations and improve segmentation accuracy. However, UNet++ may not be suitable for resource-constrained applications. DeepLab [26, 28] effectively captures global and local contextual information using atrous/dilated convolutions and multiple scales of contextual information. However, it can suffer from increased memory consumption and inference times. In their study, Wang and co-authors [12] explored the utilization of Deeplabv3+ in remote sensing for forest fire detection. They reported achieving favorable segmentation performance and efficient processing speed. Zhang et al. [31] utilized the Deeplabv3+ model for urban land use classification. They further enhanced the classification outcomes by employing a fully connected conditional random field (CRF) optimization [32]. This method employs an encoder-decoder framework to improve segmentation performance [33]. Additionally, using a context module has shown promising results in facilitating high-quality segmentation. Feature Pyramid Network (FPN) and Spatial Pyramid Pooling (SPP) methods have also been utilized for multi-scale object detection and segmentation. Still, they may encounter difficulties in handling diverse scale variations and preserving spatial resolution [34]. Dual Attention Network (DANet) introduces dual attention modules, including the position attention module and the channel attention module, to capture long-range dependencies and refine feature representations [18]. The position attention module models spatial relationships, while the channel attention module captures interdependencies between channels. Criss-cross attention (CCNet) addresses the limitation of DANet, which allows each position to attend to all positions in the feature map [17]. This mechanism captures global and local contextual information effectively. Deformable convolutional networks enhance the flexibility of standard convolutional operations by modeling the spatial sampling locations of filters [16]. This enables the network to capture deformable object structures and adapt to complex spatial variations. Furthermore, we conducted a thorough comparison with existing methods that incorporate similar techniques, such as deformable MLP and deformable patch embedding. One relevant method we compared against is the approach proposed in [35]. By including this comparison, we aimed to highlight the unique contributions and advantages of our proposed model to these existing approaches. The proposed method integrates deformable convolution with attention mechanisms after ASPP module within the UNet architecture. Deformable convolution enhances the network’s capability to capture deformable object structures, while attention mechanisms model global and local contextual information. The ASPP module captures context at multiple scales. This combination improves semantic segmentation accuracy. [36] introduces an Efficient Channel Attention (ECA) module that improves performance in deep CNNs with minimal parameters, emphasizing the importance of cross-channel interaction and avoiding dimensionality reduction. It also presents a local cross-channel interaction strategy and an adaptive kernel size selection method, beneficial for segmentation tasks. The Visual Attention Network (VAN) [37] introduces a unique convolutional neural network (CNN) backbone that integrates CNN properties and self-attention modules. The author utilizes a CNN architecture incorporating a Large Kernel Attention (LKA) module, which combines the local characteristics of CNN, the capability to capture long-range dependencies, and the spatial adaptability observed in self-attention modules similar to ViTs. Additionally, the LKA modules possess channel adaptability, a distinct attribute absent in conventional CNNs or self-attention modules used in transformers.

## 3. Materials and methods

**Fig 1:**
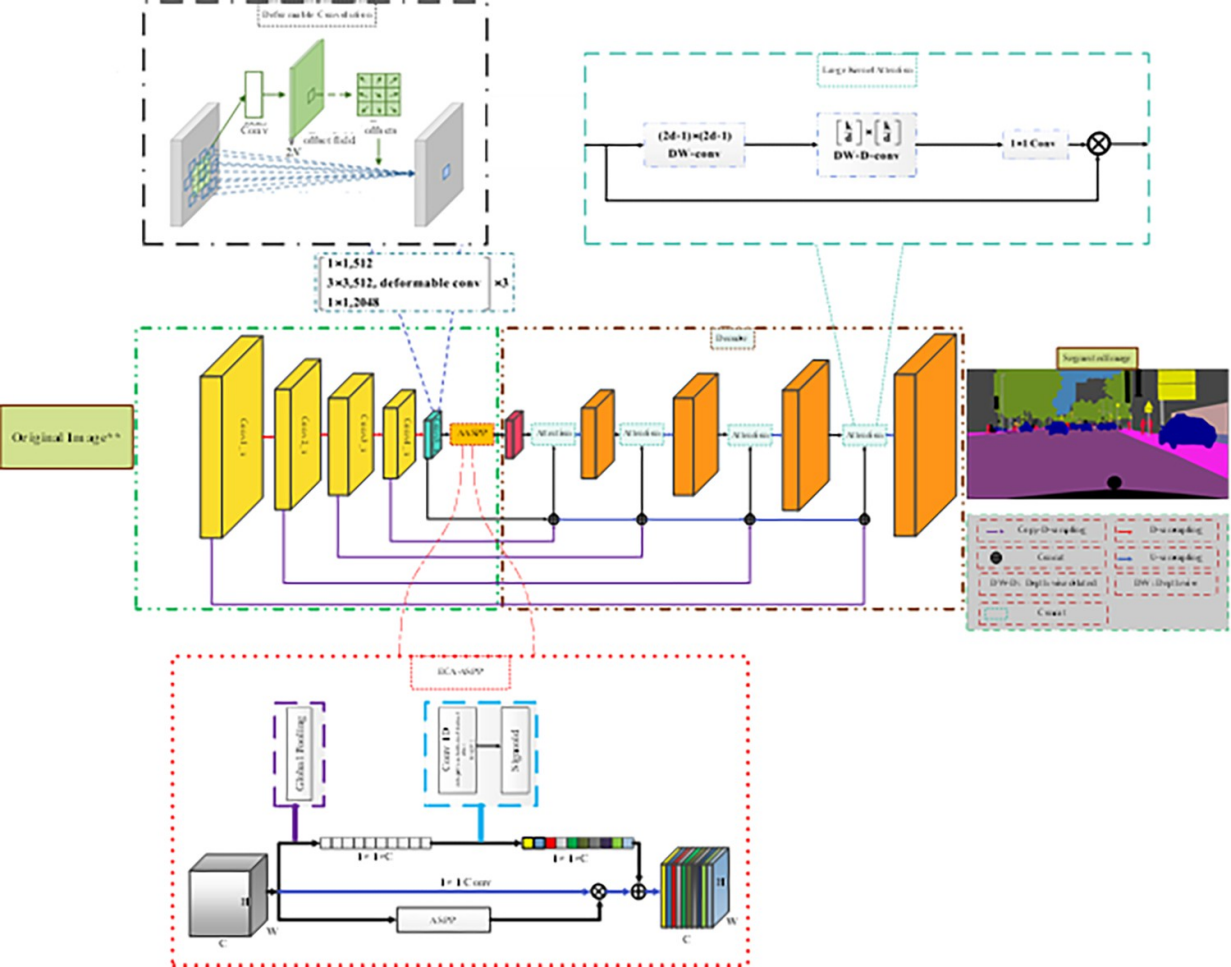
Proposed method architecture: Our proposed method combines three key components: 1) Integration of deformable convolutions within the UNet framework to enhance fine-grained details and object boundary distinction. 2) Introduction of an attention ASPP module for context modeling, utilizing attention mechanisms to capture contextual information at multiple scales. 3) Utilization of the Large Kernel Attention (LKA) module in the decoding path of the UNet to refine features and improve discrimination of object classes. ** ^Original image was taken from Cityscapes dataset which is freely available on^ [69].

This section provides detailed descriptions of the structure of proposed network and highlights how they enhance learning and feature extraction within the network, leading to improved segmentation maps. The method focuses on enhancing segmentation network performance through various architectural improvements. The encoder and decoder architectures include several advancements. An important enhancement is the utilization of deformable convolutions in the last layer of the ResNet-50 backbone. This deformable convolution operation introduces deformable sampling offsets, allowing the network to adaptively adjust the sampling locations within the receptive field. This capability enhances the model’s ability to capture fine-grained details and better handle object deformations, ultimately improving segmentation accuracy. In addition to the deformable convolutions, other architectural improvements are introduced. This work uses an enhanced version of the ResNet-50 architecture as the backbone. This improved backbone incorporates several modifications that enable the capture of hierarchical image understanding across both shallow and deep layers. By leveraging these enhancements, our approach facilitates effective multi-scale analysis, resulting in improved performance in various image analysis tasks. The architecture incorporates an attention ASPP (AASPP or ECA-ASPP) module. This module tackles the issue of semantically irrelevant high-level features (HLFs) by fusing features at the feature level. By combining the strengths of both HLFs and pixel-level features, the AASPP module bridges the gap between low-level and high-level features. As a result, it leads to improved accuracy and performance in semantic segmentation tasks. Furthermore, in the decoder path of the UNet architecture, we incorporate a large kernel attention module after the concat operation. This integration of the attention module following the concat operation enhances the capabilities of the segmentation network. Fig 1 provides an overview of the proposed model, illustrating the components and their connections. The effectiveness of the suggested IResNet-50 backbone, which incorporates deformable convolutions, and the ASPP attention module will be discussed in detail, along with comprehensive explanations of each architecture. Original image in Fig 1 was taken from dataset which is freely available on [69]. In the following subsections, we will provide a detailed explanation of each module utilized in this study. This module plays a crucial role in improving the segmentation network’s performance by focusing on relevant features.

The proposed algorithm is represented as follows:

**Algorithm 1: Algorithm of the proposed method**

1. **Input: Pre-processed image data**

2. **Output: Pixel-wise segmentation mask**

3. **Define: the UNet architecture with a ResNet50 backbone:**

  A. **Initialize the ResNet50 backbone with pre-trained weights**

  B. **Replace the last block of the ResNet50 backbone with a deformable convolution,**

**4. Define: Implement the ASPP module:**

  A. **Apply dilated convolutions at multiple rates to capture multi-scale contextual information**

  B. **Replace the concatenation module with an attention module to focus on relevant features**

**5. Implement attention modules in the decoder path:**

  A. **Replace concatenation modules with attention modules in UNet decoder architecture**

  B. **Utilize efficient channel attention to enhance discriminative power and incorporate large kernel attention to capturing long-range dependencies and contextual information**

**6. Forward pass through the network:**

  A. **Pass the input image through the encoder path**

  B. **Apply deformable convolution, ASPP module, and attention modules**

  C. **Pass the resulting features through the decoder path**

**7. Generate segmentation output:**

  A. **Apply appropriate activation functions (softmax) to obtain class probabilities**

  B. **Generate the segmentation map by assigning the class with the highest probability to each pixel**

**8. Training:**

  i. **Define the loss function (cross-entropy) for the segmentation task**

  ii. **Optimize network parameters using gradient descent-based optimization algorithms (e.g., Adam).**

**9. Evaluation:**

  i. **Evaluate the trained model on benchmark datasets**

  ii. **Calculate metrics such as Intersection over Union (IoU), accuracy, and Dice loss.**

**10. Fine-tuning and hyperparameter optimization**

  **-Explore different hyperparameter settings to optimize the proposed method’s performance**

**11. Repeat steps 6–10 until satisfactory performance is achieved.**

### 3.1. Requirement modules

#### 3.1.1. ASPP

The Atrous Spatial Pyramid Pooling (ASPP) module, originally introduced in the DeepLab paper [26], is a powerful component in CNN architectures for capturing multi-scale contextual information in an image. It aggregates features from different receptive fields of the CNN’s convolution kernel, enabling the extraction of multi-scale information. This module consists of multiple parallel branches that apply atrous spatial convolutions to the input image at different dilation rates. The dilation rate determines the spacing between the kernel elements, effectively increasing the receptive field without incurring a higher computational cost. Fig 5B provides a visual depiction of the ASPP module. Furthermore, the ASPP module allows for better feature extraction across different receptive fields since multiple dilated convolutions are concatenated through parallel branches [38].

#### 3.1.2. Deformable convolution

We describe the deformable convolution operation and provide the formulas for clarity.

**Fig 2:**
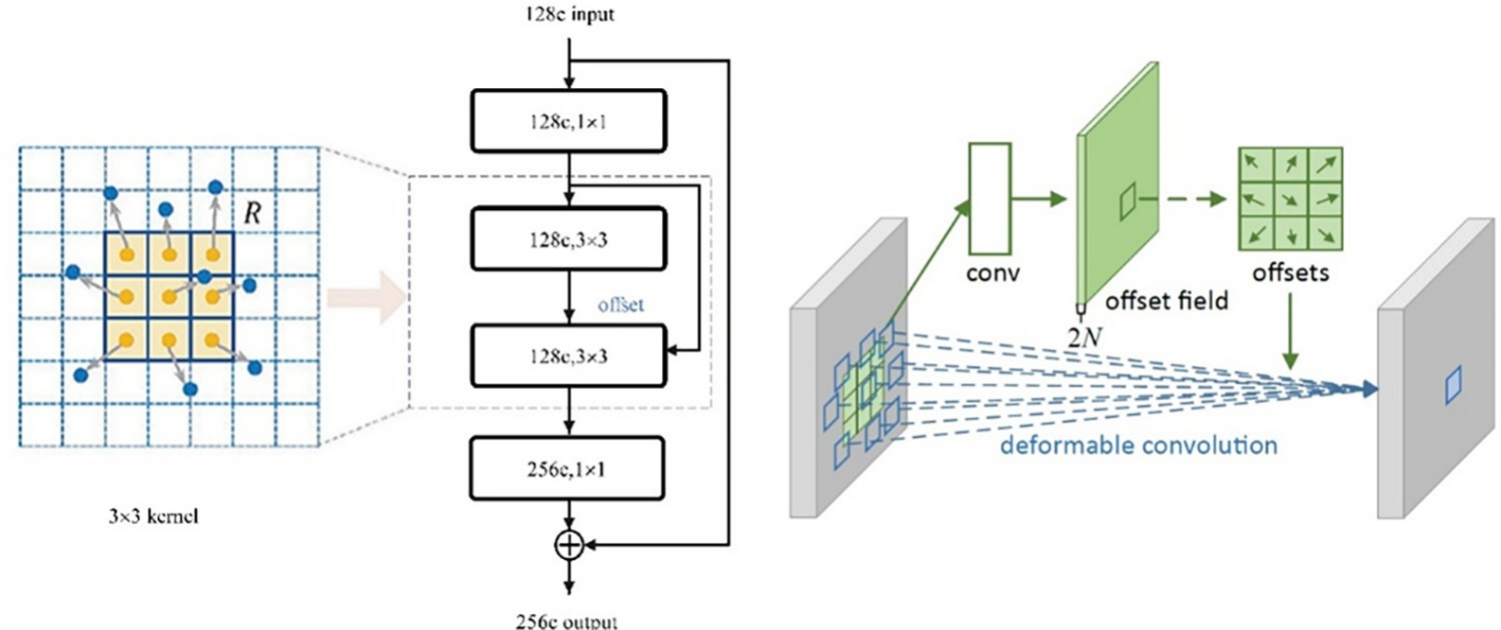
Deformable convolution [16].

Fig 2 provides a visual representation of the deformable convolution operation. Deformable convolution is an extension of the regular convolution operation, where 2D offsets are added to the sampling locations in the regular grid [16, 39]. These offsets are learned from the preceding feature maps, allowing the deformation to be conditioned on the input features in a local, dense, and adaptive manner. The resulting convolution operation is capable of capturing more intricate spatial transformations. For each location *p*_0_ on the output feature map y, the value is computed as the weighted sum of the sampled values from the input feature map x, with the weights determined by the convolution kernel w. The sampling is performed on the irregular and offset locations *p*_*n*_+Δ*p*_*n*_, where *p*_*n*_ enumerates the locations in the regular grid ℛ and Δ*p*_*n*_ represents the learned offsets. The computation can be expressed as:

R={(‐1,‐1),(‐1,0),…,(0,1),(1,1)}, (1)

$$y(p_{0})=\sum_{p_{n}\in R}{w(p}_{n})\cdot x(p_{0}+p_{n}+\Delta p_{n}),$$ (2)

To implement the deformable convolution operation, bilinear interpolation is used to compute the sampled values of x(p) at the fractional locations *p*_*n*_+Δ*p*_*n*_. Interpolation is performed using a two-dimensional kernel *G*(*q*,*p*), which is separated into two one-dimensional kernels *g*(*a*,*b*) = max (0,1−|*a*−*b*|). The computation can be expressed as:

$$x(p)=\sum_{q}G(q_{x},p_{x})\cdot G(q_{y},p_{y})\cdot x(q)$$ (3)

Where q enumerates all integral spatial locations in the input feature map x.

#### 3.1.3. Attention modules

Attention blocks improve accuracy in computer vision tasks by combining a convolutional layer with an attention module [19, 37, 40, 41]. The convolutional layer extracts feature from the input image, while the attention module selectively enhances significant features and suppresses irrelevant ones. A key component of our proposed segmentation method was the attention module.

*3*.*1*.*3*.*1*. *Efficient* channel a*ttention*. In recent years, attention mechanisms have become add to deep neural networks, aiming to improve their generalization capabilities. Several attention mechanisms have been proposed in computer vision, including Squeeze-and-Excitation Networks (SE-Net) [42], Convolutional Block Attention Modules (CBAM) [43], Bottleneck Attention Modules (BAM) [44], Global Context (GC) Nets [45], and Attention Augmented Networks (AA-Nets) [19]. However, many of these mechanisms that incorporate channel attention suffer from drawbacks such as increased complexity, dimensionality reduction, and lack of cross-channel interaction. To address these challenges, the Efficient Channel Attention (ECA) module has emerged as a lightweight and easily integrable solution [36]. It can be seamlessly incorporated into various deep convolutional neural networks, enabling efficient computation of channel attention without dimensionality reduction. Unlike other approaches like SE-Nets, CBAM, BAM, GC-Nets, and AA-Nets, which incur high computational costs, the parametric overhead introduced by ECA is minimal and proportional to the kernel size used in the 1-D convolution layer within ECA. The ECA module draws inspiration from Squeeze and Excitation Networks (SE-Nets), which are fundamental attention methods in computer vision. SE-Nets employ a three-module approach consisting of the Squeeze module, Excitation module, and Scale module. The Squeeze module reduces the tensor to a C×1×1 space using Global Average Pooling (GAP). The Excitation module, typically implemented with two fully connected layers, introduces dimensionality reduction to manage parametric overhead. ECA draws inspiration from Squeeze and Excitation Networks (SENets), which are fundamental attention methods in computer vision. SENets employ a three-module approach consisting of the Squeeze module, Excitation module, and Scale module. The Squeeze module decomposes each feature map/channel (H×W in terms of dimension) in the tensor (C×H×W) to a 1×1 space, effectively reducing the tensor to a C×1×1 space using Global Average Pooling (GAP). After the Squeeze module, the resultant tensor is passed through the Excitation module. In SENets, this involves two fully connected layers, with the first layer acting as a bottleneck defined by a reduction ratio, r. This process introduces the issue of Dimensionality Reduction (DR) to keep the parametric overhead low. SE module can be expressed as follows:

Let *X* be the input feature maps of dimensions H×W×C, where H and W are the height and width of the feature maps, and C is the number of channels.

1. Squeeze: Apply GAP to *X* to obtain a channel descriptor z of dimensions 1×1×C:

$$z(X)=\frac{1}{H\times W}\sum_{i=1}^{H}\sum_{j=1}^{W}x(i,j)$$ (4)

2. Excitation: Apply a two-layer convolution with kernel size 1 to z to obtain an attention of weights s of dimensions 1×1×C:

$$s=F_{ex}(z,C1)=\sigma (FC(\delta FC(z(X))))$$ (5)

Where *δ* is a ReLU activation function, and FC is fully connected layer. Besides, *σ* is a sigmoid activation function that scales the 1×1 output to the range [0,1], ensuring that the weights are positive and sum to 1.

3. Scaling: Apply the weights s to the original feature maps *X* to obtain the scaled feature maps *Y* of dimensions H×W×C:

Y=s⊗X (6)

Where ⊗ denotes element-wise multiplication. The scaled feature maps *Y* are then passed to the next layer in the network.

However, the authors of ECA-Net discovered that replacing the Excitation module with a simple one-to-one mapping using a single layer, where the attention weights for each channel are computed independently, outperformed SENets at a significantly lower cost. Furthermore, the authors found that using a single fully connected layer instead of two with FC, as in SE-Nets, yielded improved performance. These observations confirmed that FC negatively affects overall performance, validating the authors’ hypothesis. ECA can be represented as

$$w=\sigma ({Conv}_{k}^{1d}(y))$$ (7)

The resulting feature map utilized one-dimensional (1-D) convolutional cross-channel interaction instead of fully connected to minimize model computational complexity. If the kernel size is denoted as k, meaning that the local region of coverage can be dynamically calculated as follows:

$$k=\Psi (C)={\left|\frac{{log}_{2}(C)}{\gamma }+\frac{b}{\gamma }\right|}_{odd}$$ (8)

**Fig 3:**
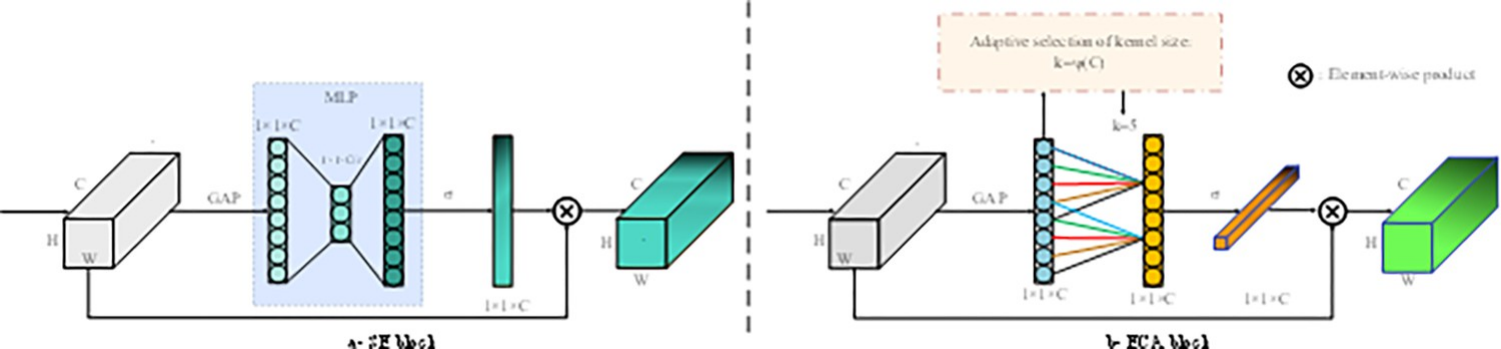
Channel attention module. SE block (a) and ECA block (b).

The symbol |.|_*odd*_ represents the nearest odd number to k. Throughout the experiments described in this paper, we obtain a clear understanding that the parameters of *γ* and *b* of the ECA module can indeed be learned during training. It was decided, however, to initialize these parameters to the default values suggested in the original paper on the ECA network. Specifically, we set *γ* to 2 and *b* to 1, in accordance with the value recommended by the authors of the paper on the ECA network. Fig 3 provides a visual representation of both the SE module and the ECA module [36].

Using GAP to extract information from each channel of the input feature map, the ECA mechanism enabled the model to prioritize essential features. The utilization of one-dimensional (1-D) convolutional cross-channel interaction in the resulting feature map, rather than a fully connected layer, enabled the model to enhance its overall performance while also minimizing computational complexity.

*3*.*1*.*3*.*2*. *Large kernel attention*. Large convolution kernels have a receptive field that can rival the self-attention mechanism. To achieve this, we can construct efficient large convolution kernels by combining depth-wise convolution, depth-wise dilated convolution, and a 1×1 convolution. These techniques allow us to maximize the benefits of self-attention and large kernel convolution while dealing with computational constraints. In our proposed network, we use the LKA (Large Kernel Attention) unit to address these challenges. The LKA unit divides a K×K convolution into three separate convolution kernels: depth-wise (DW) convolution with a size of (2d-1)×(2d−1), depth-wise dilated (DWD) convolution with a size of $\left[\frac{K}{d}]\times [\frac{K}{d}\right]$ and a dilation rate of d, and a 1×1 channel convolution. This division enables the local features modeling and the extraction of global features through depth-wise convolution dilation. Additionally, the 1×1 convolution enhances channel fusion in the feature map, improving the adaptability of the model [37, 46]. This module plays a critical role in capturing both local and global information while managing computational costs. It is particularly effective in enhancing the segmentation performance of the network by preserving fine details and modeling long-range dependencies. Moreover, it is important to note that the parameter number and floating-point operation count for the base LK convolution and its decomposition can be calculated as follows:

$${NP}_{,O}=C\times (C\times (K\times K)+1)$$ (9)

$${FLOPs}_{,O}=C\times (C\times (K\times K)+1)\times H\times W,$$ (10)

$${NP}_{,D}=C\times ((2d-1)\times (2d-1)+\frac{k}{d}\times \frac{k}{d}+C+3),$$ (11)

$${FLOPs}_{,D}=(C\times ((2d-1)\times (2d-1)+\frac{k}{d}\times \frac{k}{d}+C+3))\times H\times W,$$ (12)

Original LK convolution and Decomposed LK convolution are called *O* and *D*, respectively. If the first derivative of Eq (7) equals zero, we can identify the optimal value of d that minimizes the number of parameters.


$$\frac{d}{dd^{*}}(C{\left((2d^{*}-1\right)}^{2}+{\left(\frac{K}{d^{*}}\right)}^{2}+C+3))=0,$$ (13)

$$8d-\frac{2k^{2}}{d^{3}}-4=0$$ (14)

As a result of solving Eq (10) numerically for K = 21, the approximation of *d* of approximately 3.373 was obtained. NPs can be substantially reduced with the use of a dilation rate that is equivalent to 3, and decomposition efficiency increases as more channels are added. LKA is expressed as follows:

$$Output=[\sigma ({Conv}_{1\times 1}({Conv}_{DW}({Conv}_{DWD}(Input))))]⊗Input+Input,$$ (15)

**Fig 4:**
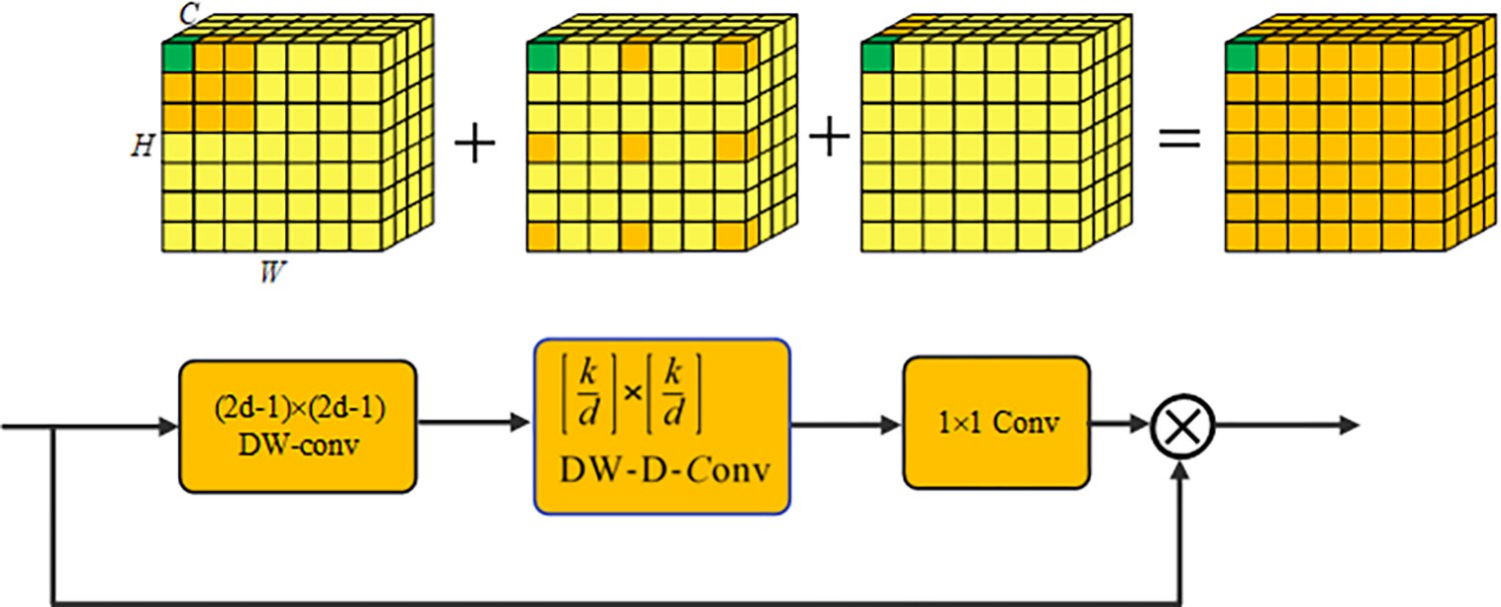
Large kernel attention module.

The LKA module output is obtained through element-wise multiplication and summation of the input feature map and the attention map. It is possible to extract extensive dependencies spanning long distances within a feature space. Furthermore, this module enables the production of an attention map with minimal computational complexity and parameter requirements [47]. Fig 4 shows that *K* = 21 represents LK convolution. It comprises three distinct components: a depth-wise convolution of size 5×5, a depth-wise dilation convolution using a 7×7 kernel with a dilation rate of 3, and a 1×1 channel convolution.

## 4. Methods

### 4.1. Replacing the concatenation operation after the ASPP module with an Efficient Channel attention module

The Pyramid Pooling Module technique captured multi-scale contextual information by pooling feature maps at different resolutions to generate a set of feature maps that could be further processed using the spatial pyramid pooling module. To this aim, a set of dilated convolutions with varying dilation rates was applied to each feature map, enabling the model to capture features at different scales. This helped the model extract relevant features more effectively and improve its performance on various computer vision tasks. The resulting perceptive feature maps were then pooled using an ECA mechanism. The ECA mechanism computed the importance score of each channel in the perceptive feature maps based on their content. The importance scores were then used to weigh the channels and combine them into a single feature map. The resulting feature maps from each level of the spatial PPM were combined using a weighted-sum operation, where the weights were the attention scores computed on each level, to produce the final feature representation, which was selectively emphasized in the informative regions at multiple scales. This allowed for better capturing of multi-scale contextual information and more robust performance in semantic image segmentation.

**Fig 5:**
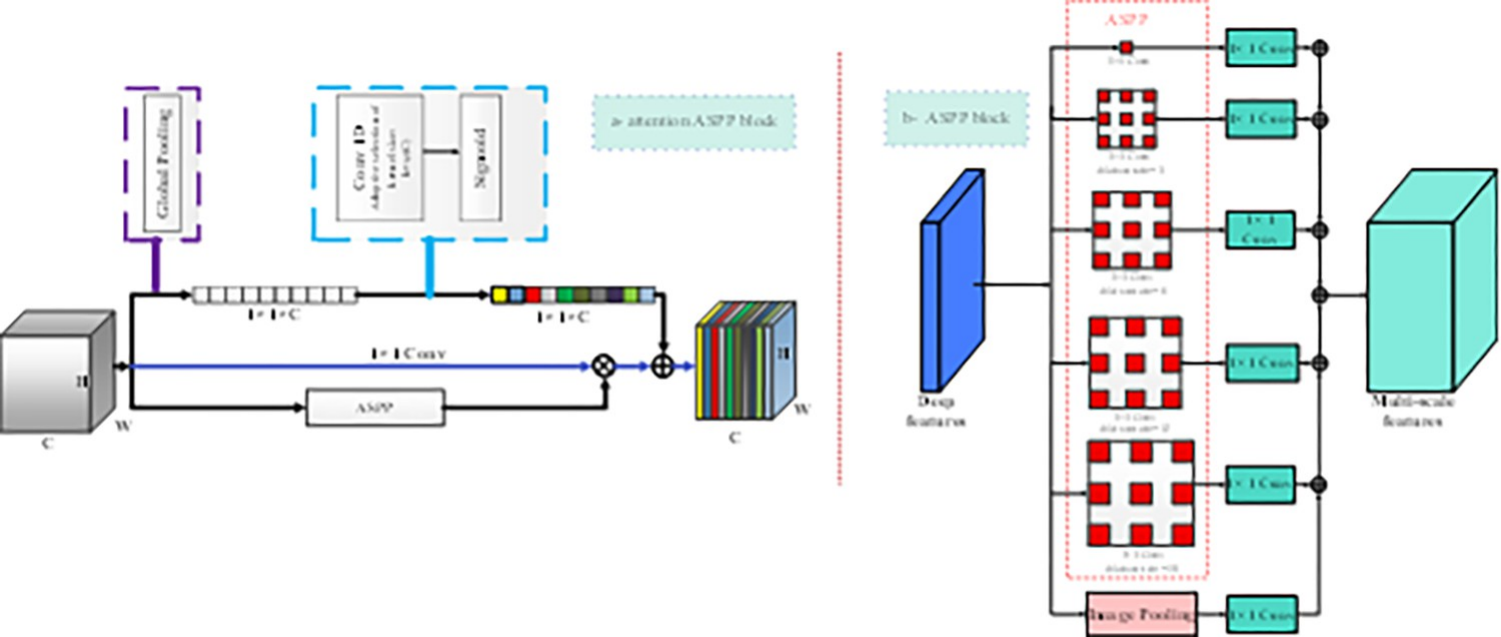
Efficient channel attention ASPP.

The ECA-ASPP might increase the risk of overfitting. The ECA-ASPP module being discussed is visualized in Fig 5A. By selectively emphasizing informative regions in feature maps at multiple scales, the module might learn to overemphasize certain regions of the image, which could lead to poor generalization performance on unseen data. This risk could be mitigated using appropriate regularization techniques, such as early stopping, data augmentation [48], and batch normalization [39, 49].

- Early stopping: Early stopping is a simple but effective regularization technique used to avoid overfitting by stopping the training process when the performance of the model on the validation set stops improving. For this purpose, a performance metric (e.g., accuracy) was selected and monitored over time.
- Data augmentation: Data augmentation facilitates increasing the training data diversity and prevents the model from overfitting to specific patterns in the training data. For this purpose, different transformations (e.g., random cropping, flipping, rotation, or scaling) were applied to the training data, which should be chosen based on the overfitting problem and the characteristics of the training data.
- Batch normalization: Batch normalization helps stabilize the distribution of activations in each network layer and prevents the model from overfitting specific patterns in the training data. To this aim, a batch normalization layer should be added after each convolutional layer in the network to normalize the activations in each batch of data and prevent the network from becoming too sensitive to specific patterns in the data.

Applying the regularization techniques mentioned in ECA-ASPP could help avoid overfitting and enhance model generalization performance. By modifying the deep neural network architecture used for semantic segmentation, the concatenation operator could be replaced by an attention module after the ASPP module. On the other hand, replacing it with an ECA module could help the network learn to selectively emphasize and weigh informative feature maps critical to the semantic segmentation task. This could improve segmentation performance by reducing the influence of less relevant features and increasing the influence of more distinct features.

### 4.2. UNet architecture based on attention

#### 4.2.1. UNet decoder based on LK attention

The UNet has served as a foundational model for several research endeavors. As a result of its ability to capture intricate object features through skip connections, it facilitates the accurate segmentation of various objects. The LK Attention-Based Decoder is a modified UNet architecture incorporating an attention-based mechanism to enhance the predictive capabilities of the UNet model. This attention-based mechanism enables the proposed model to effectively capture spatial information on the target object, yielding more precise predictions. The LKA module is used after every up-sampled module in the decoder path. LKA module details for different layers are shown in Table 1. In order to decrease the number of channels, a 1 × 1 convolution operation is then applied to the last layer. This is dependent on the number of segments in the segmentation classes. Subsequently, a softmax operation is performed to generate probability maps that delineate distinct regions associated with each class. This module is utilized in the decoder path of the UNet architecture, following the concatenation of low-level and high-level features. The LKA module is employed in the decoder path of the UNet architecture, specifically after the concatenation of low-level and high-level features, as illustrated in Fig 1.

**Table 1:** Details of large kernel attention modules.

DW Conv		DWD Conv			Equal LK Conv
Kernel	Padding	Kernel	Dilation	Padding	Kernel
(3,3)	(1,1)	(3,3)	(2,2)	(2,2)	(6,6)
(3,3)	(1,1)	(5,5)	(2,2)	(4,4)	(10,10)
(5,5)	(2,2)	(5,5)	(3,3)	(6,6)	(15,15)
(5,5)	(2,2)	(7,7)	(3,3)	(9,9)	(21,21)

#### 4.2.2. UNet architecture based on EC attention

U-Ne t architecture enhanced with Efficient Channel Attention (ECA) provides a novel approach to semantic segmentation tasks. The ECA module replaces the traditional concatenation operation in the decoder. Incorporating the ECA module into the UNet enhances the representation of features and effectively captures channel-specific dependencies, which leads to better segmentation results. By adaptively recalibrating channel-wise information, the ECA module allows the model to focus on critical features rather than suppress irrelevant ones.

#### 4.2.3. Use deformable convolution in ResNet50

Third, ResNet could be pre-trained on large datasets, e.g., ImageNet, and then fine-tuned on smaller datasets, thus enabling the transfer of learned features and significantly reducing training time. The ResNet architecture was initially proposed for image classification tasks, where detailed feature information was not always essential for accurate classification [50].

In this section, we introduce the utilization of deformable convolution as a replacement for standard convolution in the last block of the ResNet50 architecture. Deformable convolution allows for free-form deformation of the sampling grid, enabling more flexible and adaptive feature extraction.

In the context of the ResNet50 architecture, deformable convolution replaces the standard convolution operation in the last block. The offsets used in deformable convolution are obtained by applying a convolutional layer to the same input feature map. During training, the convolutional kernels for generating the output features and the offsets are learned simultaneously using backpropagation. This allows the network to identify the deformations that optimize the performance of the model. We enhance the model’s performance by adding deformable convolution to ResNet50, which makes it possible to capture complex spatial transformations. We also improve its performance on tasks that require fine-grained feature extraction. The utilization of deformable convolution in the last block of ResNet50 demonstrates its effectiveness in boosting the representational power of deep convolutional neural networks. In summary, deformable convolution presents a powerful alternative to standard convolution, enabling flexible and adaptive feature extraction. By replacing standard convolution in ResNet50’s last block with deformable convolution, we leverage its ability to capture intricate spatial transformations. This leads to improved performance in various tasks. The following formulas show the process of deformability convolution, which is clear and applicable to deep learning architectures. Table 2 provides an overview of the layer configuration.

**Table 2:** Structure of improved ResNet50.

Layer name	Output size	IResNet-50	Convolution type
Conv1_x(freeze)	224×224	7×7,64, stride2	Standard conv
Conv2_x(freeze)	112×112	$\left[\begin{array}{c} 1\times 1,64\\ 3\times 3,64\\ 1\times 1,256 \end{array}\right]\times 3$	Standard conv
Conv3_x(freeze)	56×56	$\left[\begin{array}{c} 1\times 1,128\\ 3\times 3,128\\ 1\times 1,512 \end{array}\right]\times 4$	Standard conv
Conv4_x(freeze)	28×28	$\left[\begin{array}{c} 1\times 1,256\\ 3\times 3,256\\ 1\times 1,1024 \end{array}\right]\times 6$	Standard conv
Conv5_x	14×14	$\left[\begin{array}{c} 1\times 1,512\\ 3\times 3,512\\ 1\times 1,2048 \end{array},Deformable\;conv\right]\times 3$	Deformable conv

### 4.3. Datasets and evaluation metric

The Cityscapes dataset focuses on urban street scenes and contains high-resolution images with pixel-level annotations for 19 semantic classes [51]. It provides a benchmark for evaluating semantic segmentation models in urban environments, considering factors like weather conditions and object scales. [52] is an image segmentation dataset consisting of natural scenes, such as trees and water. It is used to assess the performance and generalization ability of models in outdoor settings [53]. In addition to Cityscapes and Stanford Background, we included the DensPASS [54] and WildPASS [55] datasets in our evaluation. DensPASS captures dense urban environments, featuring high-resolution images with pixel-level annotations for semantic classes like roads, buildings, and pedestrians. WildPASS focuses on wild and natural environments, encompassing scenes like forests and mountains. These datasets allowed us to evaluate the adaptability and robustness of our model in complex urban and wild settings. Overall, our evaluation incorporated diverse datasets to assess our model’s performance and generalizability across different domains, including urban, natural, and densely populated urban environments. These datasets provided valuable insights into the practical applicability of our proposed model in various real-world scenarios.

In assessing the quality of semantic segmentation in tasks, intersection over union is an important metric. It measures the ratio between the intersection of the predicted segmentation mask, the ground truth mask, and the union of the two masks. The IoU metric ranges from 0 to 1, where a value of 1 signifies flawless segmentation. Mathematically, it can be expressed as:

$$IoU=\frac{TP}{TP+FP+FN}$$ (16)

Where TP stands for true positive (the number of correctly classified pixels), FP stands for false positive (the number of incorrectly classified pixels), and FN stands for false negative (the number of pixels that should have been classified as belonging to the class but were not). Mean Intersection over Union (mIoU) is a commonly used evaluation metric for semantic segmentation tasks, defined as the average IoU score across all classes [56].

Semantic segmentation in urban images often involves multiple classes, such as roads, buildings, pedestrians, and vehicles. Cross-entropy loss is a commonly used objective function for training models in this context.The cross-entropy loss can be defined as follows:

$$CE\;loss=-\frac{1}{N}\sum_{i=1}^{N}\sum_{c=1}^{C}Y(i,c)log(P(i,c))$$ (17)

Where N represents the total number of pixels in the image, C represents the total number of classes, *Y*(*i*,*c*) is the ground truth probability (either 0 or 1) for pixel i belonging to class c and P(i,c) is the predicted probability for pixel i belonging to class c, produced by the semantic segmentation model. The loss is calculated by summing over all pixels and all classes, penalizing the model for large deviations between the predicted probabilities and the ground truth labels. The logarithmic term log (*P*(*i*,*c*)) amplifies the error when the predicted probability is far from the ground truth value. By minimizing the cross-entropy loss during the training process, the model learns to produce accurate pixel-wise predictions, leading to improved semantic segmentation results for urban images with multiple classes [57].

### 4.4. Experimental result

In this section, we present a comprehensive evaluation of our proposed model to assess its performance, generalizability, and practical applicability. We conducted experiments on various datasets and image qualities to demonstrate the effectiveness of our approach in diverse scenarios. To evaluate the adaptability of our model, we performed an ablation study using the Stanford, Cityscapes, WildPASS, DensePASS datasets [54, 69, 70]. These datasets encompass a wide range of environmental conditions and image qualities, making them suitable for assessing the robustness of our approach. By including these datasets in our evaluation, we aimed to analyze how our model handles variations in different domains, providing insights into its generalizability. This analysis provides a comprehensive understanding of the performance of our model and its superiority over competing methods. The results obtained from the experiments demonstrate the effectiveness of our proposed model in handling diverse scenarios and image qualities. We observed significant improvements in terms of accuracy, robustness, and adaptability compared to baseline models. These findings support the practical applicability of our approach in real-world scenarios and underline its potential for various computer vision tasks, including panoramic semantic segmentation.

#### 4.4.1. Ablation study

Table 3 compares different modules and their combinations in the UNet-based models. The modules evaluated include D-Conv (last layer-ResNet50), ECA-ASPP (AASPP), ECA (decoder), LKA (decoder), and ASPP. The proposed method, which combines various modules, achieves high performance across different configurations. It demonstrates the effectiveness of using Improved ResNet50, ECA-ASPP, ECA (decoder), and AASPP in the UNet architecture. The table provides valuable insights for selecting suitable modules for optimizing UNet-based semantic segmentation models. An ablation study compared the performance of three different semantic segmentation models shown in Tables 4 and 5.

**Table 3:** Different modules used in the proposed UNet-based architectures.

**Module**	**UNet-R** ^*^	**UNet-IR** ^**^	**UNet-R-AASPP** ^***^	**UNet-IR-AASPP**	**UNet-R-LKA**	**UNet-IR-LKA**
**D-Conv(last layer-ResNet50)**	×	✓	×	✓	×	✓
**ECA-ASPP(AASPP)**	×	×	✓	✓	×	×
**ECA(decoder)**	×	×	×	×	×	×
**LKA(decoder)**	×	×	×	×	✓	✓
**ASPP**	✓	✓	×	×	✓	✓
	**UNet-R-ECA**	**UNet-IR-ECA**	**UNet-R-AASPP-ECA**	**UNet-IR-AASPP-ECA**	**UNet-R-AASPP-LKA**	**UNet-IR-AASPP-LKA** **Proposed method**
**D** ^****^ **-Conv (last layer)**	×	✓	×	✓	×	✓
**ECA-ASPP**	×	×	✓	✓	✓	✓
**ECA(decoder)**	✓	✓	✓	✓	✓	✓
**LKA(decoder)**	×	×	×	×	✓	✓
**ASPP**	✓	✓	×	×	×	×

* R = ResNet50

** IR = Improved ResNet50

*** Attention ASPP

****D: Deformable

**Table 4:** Effect of additional modules on segmentation performance: Ablation study results in Stanford dataset.

**class**	**UNet-R**			**UNet-IR**			**UNet-R-AASPP**		
	**IoU**	**Dice**	**Acc**	**IoU**	**Dice**	**Acc**	**IoU**	**Dice**	**Acc**
**Sky**	80.36	89.11	86.81	80.02	88.90	95.32	88.88	94.113	95.4
**Tree**	62.67	77.05	75.31	66.92	80.18	74.21	67.06	80.283	76.84
**Road**	70.37	82.61	82.76	79.99	88.88	85.7	88.68	94.000	95.09
**Grass**	65.31	79.02	80.33	70.79	82.90	81.81	74.37	85.301	93.09
**Water**	52.36	68.73	78.61	37.52	54.57	81.92	80.48	89.184	88.26
**Bldg**	70.05	82.39	81.02	77.07	87.05	89.19	80.34	89.098	88.17
**Mntn**	21.52	35.42	25.01	41.64	58.80	40.38	26.92	42.420	31.39
**Fg-obj**	59.5	74.61	70.72	62.84	77.18	77.83	70.37	82.608	85.61
**Mean**	60.27	73.62	72.57	64.60	77.31	78.30	72.14	82.13	81.73
**Class**	**UNet-IR-AASPP**			**UNet-R-LKA**			**UNet-IR-LKA**		
	**IoU**	**Dice**	**Acc**	**IoU**	**Dice**	**Acc**	**IoU**	**Dice**	**Acc**
**Sky**	88.81	94.073	94.3	89.16	94.27	93.9	91.26	95.43	**96.55**
**Tree**	70.97	83.020	84.76	70.81	82.91	83.65	75.95	86.33	81.89
**Road**	88.58	93.944	95.67	89.15	94.26	94.44	90.67	95.11	**97.56**
**Grass**	77.95	87.609	89.72	78.87	88.19	88.26	88.74	94.03	**96.24**
**Water**	78.54	87.980	83.93	78.53	87.97	83.5	**92.55**	96.13	**96.53**
**Bldg**	79.25	88.424	88.49	79.42	88.53	89.12	84.84	91.80	**93.89**
**Mntn**	50.25	66.889	58.7	54.1	70.21	66.87	61.25	75.97	68.61
**Fg-obj**	72.51	84.065	81.31	72.06	83.76	85.03	76.99	87.00	85.15
**Mean**	75.86	85.75	84.61	76.51	86.26	85.60	82.78	90.22	89.55
**Class**	**UNet-R-ECA**			**UNet-IR-ECA**			**UNet-R-AASPP-ECA**		
	**IoU**	**Dice**	**Acc**	**IoU**	**Dice**	**Acc**	**IoU**	**Dice**	**Acc**
**Sky**	88.93	94.14	94.3	91.49	95.56	94.63	89.79	94.62	96.06
**Tree**	70.96	83.01	84.76	76.35	86.59	87.79	73.2	84.53	80.99
**Road**	88.58	93.94	95.67	90.09	94.79	97.32	89.91	94.69	95.53
**Grass**	77.95	87.61	89.72	85.65	92.27	93.47	83.21	90.84	95.76
**Water**	78.58	88.01	83.93	88.71	94.02	95.28	86.94	93.01	91.64
**Bldg**	79.25	88.42	88.49	82.48	90.40	87.73	83.16	90.81	91.5
**Mntn**	50.25	66.89	58.7	57.88	73.32	80.46	39.21	56.33	42.35
**Fg-obj**	70.63	82.79	81.31	73.28	84.58	85.47	73.85	84.96	87.21
**Mean**	75.64	85.60	84.61	80.74	88.94	90.27	77.41	86.22	85.13
**Class**	**UNet-IR-AASPP-ECA**			**UNet-R-AASPP-LKA**			**UNet-IR-AASPP-LKA**		
	**IoU**	**Dice**	**Acc**	**IoU**	**Dice**	**Acc**	**IoU**	**Dice**	**Acc**
**Sky**	91.36	95.48	95.38	88.9	94.12	94.26	**91.91**	**95.78**	95.76
**Tree**	78.39	87.89	86.39	76.03	86.38	**88.97**	**79.94**	**88.85**	88.65
**Road**	89.26	94.33	97.06	90.2	94.85	95.54	**90.86**	**95.21**	97.51
**Grass**	90.71	95.13	95.53	82.66	90.51	90.18	**90.91**	**95.24**	95.72
**Water**	92.17	95.93	96.05	78.86	88.18	86.59	91.92	95.79	96.64
**Bldg**	85.35	92.10	92.23	81.11	89.57	87.01	**87.15**	**93.13**	92.99
**Mntn**	68.65	81.41	77.7	65.59	79.22	71.05	**75.2**	**85.84**	**81.45**
**Fg-obj**	75.25	85.88	85.73	72.42	84.00	86.43	**78.42**	**87.90**	**87.34**
**Mean**	83.89	91.02	90.76	79.47	88.35	87.50	**85.79**	**92.22**	**92.01**

**Table 5:** Effect of additional modules on segmentation performance: Ablation study results in Cityscapes dataset.

**Method**	**UNet-R**	**UNet-IR**	**UNet-R-AASPP**	**UNet-IR-AASPP**	**UNet-R-LKA**	**UNet-IR-LKA**
**mIOU**	68.27	71.47	72.25	74.23	78.8	79.41
**mACC**	80.54	79.22	79.26	82.14	86.85	87.01
**mDice**	81.14	83.36	83.89	85.21	88.14	88.52
**Method**	**UNet-R-ECA**	**UNet-IR-ECA**	**UNet-R-AASPP-ECA**	**UNet-IR-AASPP-ECA**	**UNet-R-AASPP-LKA**	**UNet-IR-AASPP-LKA**
**mIOU**	75.63	77.12	79.09	80.16	81.35	**82.25**
**mACC**	83.96	84.82	86.57	87.79	88.1	**88.74**
**mDice**	86.12	87.08	88.32	88.99	89.72	**90.26**

Upon reviewing the provided data, it is evident that the UNet-IR-AASPP-LKA model is indeed the best performer among the proposed methods. Across various semantic classes, it consistently achieves higher IoU, Dice, and accuracy scores compared to other models. In the Sky class, UNet-IR-AASPP-LKA achieves an IoU of 88.9 and a Dice coefficient of 94.12, indicating its ability to accurately segment sky regions. Similarly, in the Tree class, it achieves competitive scores with an IoU of 76.03 and a Dice coefficient of 86.38, demonstrating its proficiency in segmenting trees. The UNet-IR-AASPP-LKA model also performs well in other classes such as Road, Grass, Water, and Building, consistently achieving high scores for IoU, Dice, and accuracy. Its overall performance, as indicated by the mean scores, is significantly better compared to the other reported models. It is important to note that the UNet-R-AASPP-ECA model also shows promising results, particularly in the Water class, where it outperforms other models by a significant margin. However, the UNet-IR-AASPP-LKA model consistently demonstrates superior performance across multiple classes. In conclusion, the UNet-IR-AASPP-LKA model emerges as the most effective among the proposed methods, showcasing its capability to accurately segment various objects and achieve high-quality semantic segmentation results. In Table 4, the results of the ablation study on the Stanford dataset are illustrated. A comparative analysis of the impact of additional modules is presented.

**Fig 6:**
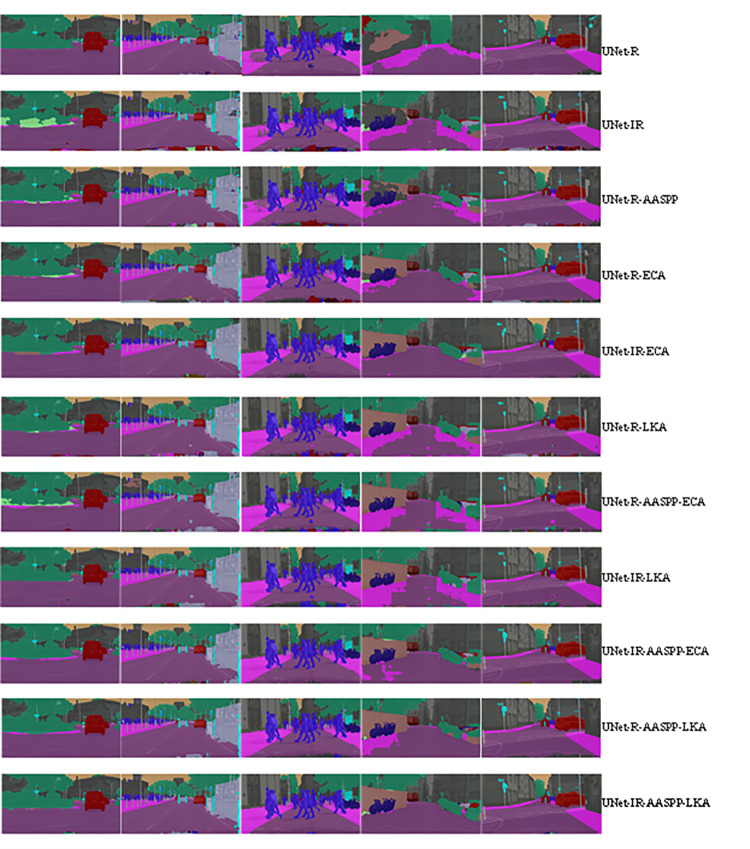
Ablation study results: Comparative analysis of additional modules on segmentation performance in Cityscapes dataset. (Original image was taken from Cityscapes dataset which is freely available on [69]).

**Fig 7:**
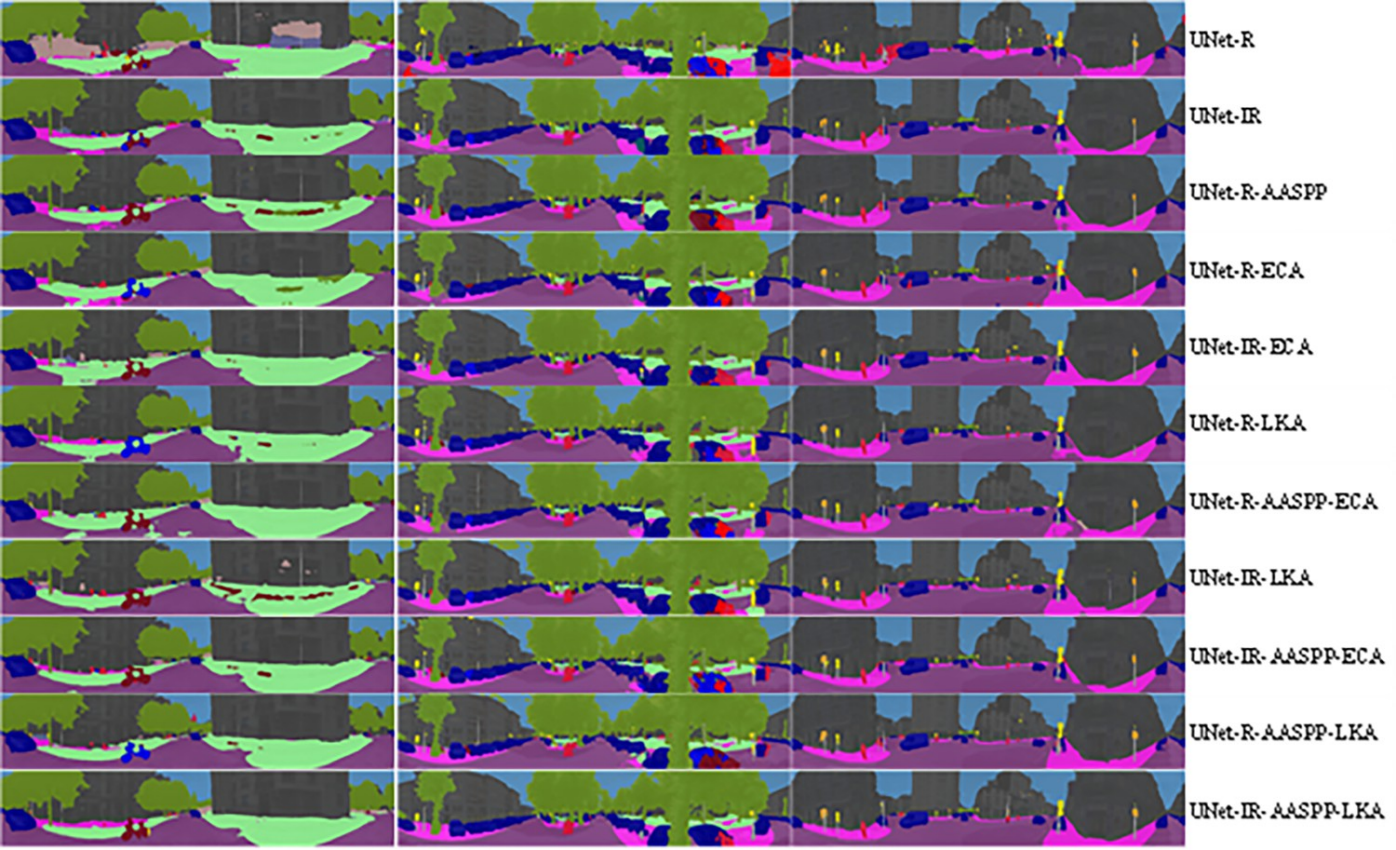
Ablation study results: Comparative analysis of additional modules on segmentation performance in WildPASS and DensPASS datasets. (Original image was taken from WildPASS and DensPASS datasets which are freely available on [54, 70]).

The ablation study results presented in Fig 6 [69] and Fig 7 [54, 70] provide a comparative analysis of the impact of additional modules on the segmentation performance of the model. Fig 6 shows the results for the Cityscapes dataset [69], while Fig 7 presents the findings for the WildPASS and DensPASS datasets [54, 70]. Both figures illustrate the comparative segmentation performance when incorporating various additional modules into the base model. The Cityscapes dataset [69] and the WildPASS and DensPASS datasets [54, 70] are publicly available and were used as the source for the images presented in these figures.

Based on an ablation study of the Cityscapes dataset, Table 5 presents the results of exploring the impact of additional modules on segmentation performance. It has been demonstrated incorporating advanced modules, such as ASPP, LKA, and ECA, consistently improves segmentation metrics compared to the baseline UNet model. As a result of the inclusion of these modules, mIOU, mACC, and mDice scores were markedly improved. UNet with IR -AASPP-LKA shows the highest performance, with a mIOU of 82.25, mACC of 88.74, and mDice of 90.26. These results highlight the effectiveness of integrating specific modules to enhance the UNet model’s segmentation performance on the Cityscapes dataset, demonstrating the importance of advanced techniques in achieving superior segmentation results.

#### 4.4.2. Results comparison

The performance of various semantic segmentation methods was compared on different datasets in Tables 6–8. The "Proposed method" with the IResNet50 backbone consistently achieved high IOU scores, indicating its superior performance in accurately segmenting objects and regions. It outperformed other models on the Cityscapes, DensPASS, and WildPASS datasets, showcasing its versatility across different environmental scenes. Additionally, the proposed method exhibited exceptional performance in segmenting water regions on the Stanford dataset. These findings provide valuable insights for researchers and practitioners in selecting effective models for scene-understanding tasks.

**Table 6:** Performance comparison of semantic segmentation methods on Cityscapes, DensPASS.

method	backbone	Cityscapes	DensPASS
**Fast-SCNN [58]**	**Fast-SCNN**	69.1	24.6
**FCN-32 [27]**	**VGG-16**	62.74	-
**S-FPN [60]**	**ResNet101**	75.8	28.8
**FPN [60]**	**VGG-16**	67.87	-
**PSPNet [62]**	**ResNet50**	78.6	29.5
**DANet [18]**	**ResNet101**	79.3	28.2
**UNet++ [30]**	**ResNet50**	70.96	-
DeepLabV3#layer 1^*^ **[16]**	**ResNet101**	73.5	30.41
**DeepLabV3+ [13]**	**ResNet101**	80.9	32.5
**Segformer-B1 [66]**	**Segformer-B1**	78.5	38.5
**Trans4PASS-S [35]**	**Trans4PASS-S**	**81.1**	**44.8**
**Proposed method**	**IResNet50**	**82.25**	**44.9**

* use deformable conv in #1 layer of backbone

**Table 7:** Performance comparison of semantic segmentation methods on Cityscapes.

method	WildPASS
**Fast-SCNN [58]**	28.5
**SegNet [59]**	25.7
**BiSeNet [61]**	27.7
**PSPNet [62]**	41.4
**CGNet [45]**	30.4
**DANet [18]**	47.2
**SwiftNet [63]**	38.2
**ENet [64]**	23.8
**ERF-PSPNet [65]**	58.4
**omni-supervised** **(ERF-PSPNet) [55]**	**66.8**
**ECANet [55]**	60.2
**Proposed method**	**67.1**

**Table 8:** Class-wise IOU comparison of segmentation models on the Stanford dataset.

Method	backbone	Sky	Tree	Road	Grass	Water	Building	Mountain	Foreground	mIoU
**FCN [27]**	**VGG-16**	78.98	49.03	71.64	65.33	43.73	47.17	11.9	40.1	50.99
**UNet [29]**	**ResNet50**	85.68	64.62	85.13	69.06	60.08	74.96	6.03	61.58	63.39
**FPN [60]**	**ResNet50**	88.93	65.64	83.57	75.93	69.12	74.56	20.83	62.59	67.65
**Pspnet [62]**	**ResNet50**	79.99	54.29	81.49	65.99	50.93	70.47	12.12	55.78	58.88
**CCNet [17]**	**ResNet101**	89.4	72.59	87.36	77.58	72.83	80.09	49.23	66.74	74.48
**Unet++ [30]**	**ResNet50**	88.65	65.5	85.87	75.61	79.3	78.31	17.68	66.34	69.66
**DeepLabV3 [67]**	**ResNet50**	84.88	64.56	85.9	73.59	75.06	75.15	41.64	59.38	70.02
**DeepLabV3+ [13]**	**ResNet50**	87.1	68.56	87.97	79.84	77.43	78.03	35.8	68.72	72.93
**DANet [18]**	**ResNet101**	89.42	72.25	87.32	77.48	72.74	80.07	48.68	66.96	74.37
**DLv3+ &LoAd [68]**	**ResNet101**	89.51	72.93	87.75	77.94	73.93	80.91	50.15	67.58	75.09
**Trans4PASS-S [35]**	**Trans4PASS-S**	**92.37**	**85.48**	89.02	78.44	87.99	86.73	36.86	**78.73**	79.45
**Proposed method**	**IResNet50**	91.91	79.94	**90.86**	**90.91**	**91.92**	**87.15**	**75.2**	78.42	**85.79**

In Table 6, we observe that the "Proposed method" utilizing the IResNet50 backbone with LK attention achieves the highest IOU score of 82.25 on the Cityscapes dataset. This indicates its superior performance in accurately segmenting objects and regions within urban scenes. Among the other models, Trans4PASS-S with the Trans4PASS-S backbone achieves the second-highest IOU score of 81.1. These results highlight the effectiveness of both the proposed method and the Trans4PASS-S model in urban scene understanding. Moving to the DensPASS dataset, we observe that the "Proposed method" and Trans4PASS-S once again showcase remarkable performance, with IOU scores of 44.9 and 44.8, respectively. These scores indicate the ability of these models to handle the complex and dense scenarios present in the DensPASS dataset. It is worth noting that the proposed method achieves the highest IOU score on both the Cityscapes and DensPASS datasets, demonstrating its versatility and effectiveness across different environmental scenes.

Table 7, presents the performance comparison on the WildPASS dataset. Among the models evaluated, the "Proposed method" achieves the highest IOU score of 66.7 on the WildPASS dataset, showcasing its competence in handling challenging and diverse natural environments.

Table 8 provides a class-wise IOU comparison of segmentation models on the Stanford dataset. It reveals that the "Proposed method" with the IResNet50 backbone achieves remarkable IOU scores across various classes, including Sky, Tree, Road, Grass, Water, Building, Mountain, and Foreground. Notably, it achieves the highest IOU score of 91.92 for the Water class, indicating its ability to accurately segment water regions within the scene.

In summary, the comparative analysis of semantic segmentation models demonstrates the effectiveness of the "Proposed method" with the IResNet50 backbone across multiple datasets and environmental conditions. It consistently achieves high IOU scores, indicating its superior performance in accurately segmenting different objects and regions. These findings provide valuable insights for researchers and practitioners in selecting suitable models for environmental scene understanding tasks.

**Fig 8:**
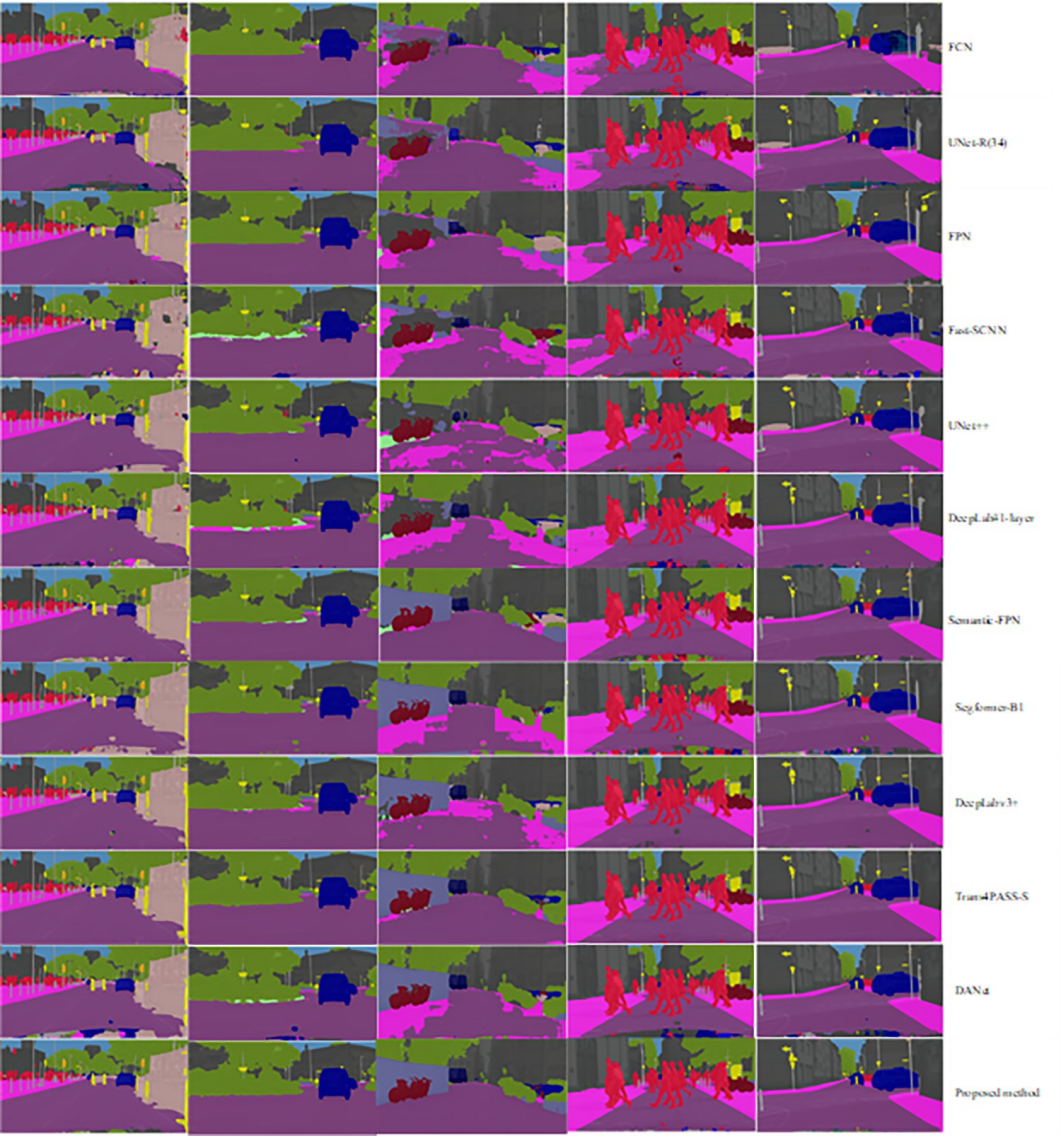
Visual comparison of original [69] and segmented images using state of the art (FCN [27], UNet-R(34) [29], FPN [60], Fast-SCNN [58], UNet++ [30], DeepLabV3#layer 1 [16], S-FPN [60], Segformer-B1 [66], DeepLabV3+ [13], Trans4PASS-S [35], DANet [18]) and proposed method in the Cityscapes dataset. (Original image was taken from Cityscapes dataset which is freely available on [69]).

Fig 8 displays a comparison between the original images and segmented images generated by various existing models and the proposed method. A challenging benchmark for semantic image segmentation is provided by the Cityscapes dataset composed of urban scenes.

## 5. Conclusion

A comprehensive study on enhancing the performance of the UNET architecture for image segmentation tasks was presented in this paper. The proposed approach utilized a combination of techniques, including integrating the ResNet50 backbone with a modified last block employing deformable convolution. In addition, an ASPP attention module, such as ECA or LKA, has been integrated into the UNet decoder path. This study explored the impact of different ablation settings and resulted in 12 separate conclusions based on extensive experimentation and analysis. In particular, a model that used the UNet with an enhanced ResNet50 backbone and integrated AASPP module into LKA decoder path has been identified as one of the best performance models. The results demonstrated superior segmentation performance compared to well-known models, as evidenced by the presented visual results. As far as future work is concerned, there are some possibilities for further improving performance. One direction is investigating alternative architecture modifications beyond those examined in this study. Exploring different backbone networks could also be beneficial, as it may provide additional insights into improving segmentation accuracy. Furthermore, incorporating novel attention mechanisms or exploring the combination of multiple attention modules could offer potential improvements. Overall, this research provides a solid foundation for future investigations in image segmentation. It aims to advance state-of-the-art performance through innovative architectural modifications and network enhancements.

## Supporting information

S1 Data (DOCX)
