# Supplementary material for: Advancing semantic segmentation: Enhanced UNet algorithm with attention mechanism and deformable convolution
Source: PLoS One. 2025 Jan 16;20(1):e0305561. doi: 10.1371/journal.pone.0305561 (PMC11737789; doi:10.1371/journal.pone.0305561)
Supplement: S1 Data — (DOCX) [file pone.0305561.s001.docx]

Dear Editor,

Thank you for your request regarding the data availability for our submission. We can confirm that our submission contains the minimal data set required to replicate the results of our study.

Specifically, we utilized the following publicly available datasets in our work:

1. Cityscapes Dataset - Semantic Understanding of Urban Street Scenes (https://www.cityscapes-dataset.com/)

2. Stanford 2D-3D Dataset (http://dags.stanford.edu/data/iccv09Data.tar.gz)

3. panoramic Dataset(DensePASS and WildPASS) (https://github.com/elnino9ykl/WildPASS?tab=readme-ov-file and https://github.com/chma1024/DensePASS)

For data preprocessing, we normalized the images using mean=[123.675, 116.28, 103.53] and std=[58.395, 57.12, 57.375]. We also applied various data augmentation techniques including RandomResize, RandomCrop, and RandomFlip.

Additionally, we used the following optimizer parameters in our training:

- Optimizer: SGD

- Learning rate: 0.01

- Momentum: 0.9

- Weight decay: 0.0005

Please let us know if you require any additional information. We are happy to provide the minimal data set necessary to replicate our findings.

Sincerely,
